# Evaluation of Immunization Knowledge, Practices, and Service-delivery in the Private Sector in Cambodia

**Published:** 2008-03

**Authors:** Sann Chan Soeung, John Grundy, Cheng Morn, Chham Samnang

**Affiliations:** 1 National Immunization Program, Ministry of Health, Cambodia; 2 Mekong Program, Nossal Institute for Global Health, University of Melbourne, 187 Grattan Street, Carlton, Victoria 3010, Australia (Cambodia Office: House 194, Street 63, Phnom Penh, Cambodia); 3 Immunization Program at PATH Cambodia, c/o Box 1684, Phnom Penh, Cambodia

**Keywords:** Evaluation studies, Health services, Health-sector reforms, Health systems, Immunization, Private sector, Quality of care, Standards, Cambodia

## Abstract

A study of private-sector immunization services was undertaken to assess scope of practice and quality of care and to identify opportunities for the development of models of collaboration between the public and the private health sector. A questionnaire survey was conducted with health providers at 127 private facilities; clinical practices were directly observed; and a policy forum was held for government representatives, private healthcare providers, and international partners. In terms of prevalence of private-sector provision of immunization services, 93% of the private inpatient clinics surveyed provided immunization services. The private sector demonstrated a lack of quality of care and management in terms of health workers’ knowledge of immunization schedules, waste and vaccine management practices, and exchange of health information with the public sector. Policy and operational guidelines are required for private-sector immunization practices that address critical subject areas, such as setting of standards, capacity-building, public-sector monitoring, and exchange of health information between the public and the private sector. Such public/private collaborations will keep pace with the trends towards the development of private-sector provision of health services in developing countries.

## INTRODUCTION

In 1993, Cambodia (population 14 million) emerged from a lengthy period of civil conflict that ended when United Nations-sponsored general elections were held. Since then, the country has entered an era of economic and social development. Significant developments occurred within the two main social sectors of health and education. Health-sector reform, begun in 1996, led to the establishment of national health institutions, 24 provincial health departments, 76 operational health districts, and more than 930 health centres, most of which serve the rural communities that comprise 80% of the country's total population. As a result of the developing market economy and rapid urbanization, a professional private health sector emerged in the middle of the 1990s with a range of studies indicating that it is expanding at a rapid rate (1,2). Today, two-thirds of the population turn first to the private sector or non-medical sector for medical care (3).

Established in 1986 with the technical assistance of the United Nations Children's Fund (UNICEF), the National Immunization Program (NIP) manages the immunization of an annual cohort of 372,000 infants aged less than one year (2006) against seven diseases and also against tetanus for women of childbearing age. Demographic health surveys conducted in 2000 and 2005 have seen a rise in immunization coverage rate of diphtheria, pertussis and tetanus vaccine (DPT3) in children aged 12 months. The rates of coverage have increased from 43% in 2000 to 76% in 2005, with associated sharp declines in reported vaccine-preventable diseases (3). Cases of neonatal tetanus have reduced from 169 in 2002 to 68 in 2005 (decline in incidence from 0.05 to 0.02 per 1,000 births). Suspected cases of measles have been reduced from 3,640 in 2001 to 264 in 2005 (decline in incidence from 289 to 19 per million population), and the coverage of measles immunization has increased from 41% in 2000 to 70% in 2005. This programme is administered through the state health system, with most service-delivery taking place at the primary-level health centres through outreach services to the 13,000 villages of Cambodia.

A wide definition of the private sector can be described as “comprising all providers who exist outside the public sector, whether their aim is philanthropic or commercial” (4). For the purpose of the collaboration discussed in this paper, the private sector is referred to as the private, for-profit medical and professional health sector.

Results of recent research in Cambodia have indicated a high prevalence of private, for-profit provision of medical care, some of which has been reported to be of dubious quality. A survey of 198 private doctors in Phnom Penh indicated that more than half of all consultations resulted in the inappropriate prescription of drugs (2). Results of a different study indicated that case management by 108 private doctors included high rates of incorrect diagnoses and improper treatment of acute respiratory infections and diarrhoea in children (1).

Evidence suggests that the private professional healthcare sector is now larger than the public healthcare sector. The province of Kampong Cham with a population of 1.7 million, for example, maintains a base of 153 private facilities, half of which are licensed. Public-sector services in the province are provided from 141 facilities (130 public-health centres and 10 referral hospitals). Therefore, more private-sector than public-sector healthcare outlets exist in this province. This is of particular significance because the province has the largest population base in the country.

To build on experience in both public-sector and private-sector healthcare settings, some national programmes, such as those for tuberculosis and reproductive health, have recently developed collaboration models. Furthermore, the Ministry of Health (MoH) identified public-private collaboration as a key strategy in its Health Sector Strategic Plan (5). The role of public institutions in regulating elements of private practice was further illustrated by the Royal Government of Cambodia's enactment of the Health Law in 2000, which stipulates the conditions under which private-sector providers can operate and identifies the roles and responsibilities of MoH for monitoring pharmaceutical practice and imports, including vaccines (6).

A wide-ranging immunization review, conducted by international partners in 2005, found evidence of low adherence to quality standards in the private sector (7). Given these findings and following on policies enacted through the Health Law, the MoH asked the NIP and its partners to explore potential models of collaboration between the public and the private sector aimed at increasing access of women and children to quality immunization services.

The NIP entered into a partnership with PATH and local research consultant firm—Domrei Consulting—to conduct a research study on immunization services provided in the private sector. Based on this research and follow-up consultation activities, this paper describes the following: (a) The scope of immunization practices among private practitioners in defined settings; (b) An assessment of the quality of care in relation to private-sector immunization practices; and (c) Existing models and potential opportunities for service collaboration between the public and the private sector.

## MATERIALS AND METHODS

### Sources of information

The three main sources of information included: a survey questionnaire accompanied by direct observation of clinical practices, a policy forum with public- and private-sector participation, and review of national and international literature.

#### Research survey

Four teams collected information in four cities in Cambodia: Phnom Penh, Kampong Cham, Battambang, and Sihanoukville. Cambodia has six major cities. The fifth city (Siem Reap) was not included because of the expected lower prevalence of private clinics in this city, and the sixth (Kandal City) was not included because of proximity to the capital city Phnom Penh. Four teams surveyed 127 private facilities, which included 28 clinics providing inpatient services, 73 cabinets providing outpatient services, 17 pharmacies, and 9 laboratories. The survey was conducted during 13–16 July 2005.

The MoH licensed 69% of these facilities. Supply of vaccine stock was directly observed in 81% of the facilities, and patient records were checked in 83% of the facilities. Respondents included the providers (nurses and doctors) of immunization services at the private facilities. There were two refusals from the facilities to participate in the survey. Public facilities or NGO provider services were not included in the survey sample.

The survey questionnaire was designed to meet three objectives: (a) identify the scope of immunization practices, (b) assess the quality of care, and (c) identify opportunities for collaboration between the public and the private sector. The questionnaire was divided into three sections. A PATH interviewer administered it to facility providers with the consent of the facility manager in the presence of one provincial health department staff member. In Section 1 of the questionnaire, the interviewer recorded information about the first survey objective 1. In Section 2, the interviewer recorded information about the third survey objective 3, i.e. willingness to collaborate with the public sector. This section preceded the questions on supply and storage of vaccines and quality of care to avoid leading the respondents. Section 3 was organized into themes of (a) supply of vaccines (one series of questions for each vaccine), (b) storage of vaccine, and (c) quality of immunization services. At the end of Section 3, the interviewers asked to see immunization records and stock of vaccines and recorded their observations on the questionnaire.

#### Policy forum

A policy forum was conducted in Phnom Penh in November 2005 on the subject of public-private sector collaboration for immunization. Participants included private-sector clinical managers, representatives of non-governmental organizations (NGOs), public-health officials, and immunization managers from the public sector (in total 50 participants). The objectives of the forum were to present findings of research and to reach consensus on policy and framework for the implementation of immunization quality standards. Data were collected through open discussions in relation to research findings and to recommendations from participants towards developing guidelines for private-sector immunization practices.

#### Literature review

Literature was collected nationally and included public-health law and national health policy and plans and also included selected published and unpublished research reports on private-sector healthcare in Cambodia. A review of international literature on involvement of the private sector in immunization service provision and management was also conducted.

### Ethics

Ethical clearance waivers were obtained through the institutional ethics committee of PATH. The Ministry of Health (NIP) also waived the requirement for formal ethical committee review, on the basis that the research involved internal evaluation of health programme. Consent was obtained for interviewing private-sector practitioners through sending of letters to all private-sector managers inviting them to participate in the study one week before visiting the clinic for interview and observation. Of the 127 facilities, there were two refusals.

### Sampling and study limitations

Private clinics were selected based on researchers’ assessments of the feasibility of these clinics entering into a partnership with the Government, i.e. the largest clinics with the highest numbers of infant deliveries, and based on their size, i.e. those most likely to provide immunization services. The intent of this purposeful selection was to assist to identify early adopters of a collaboration strategy with the public sector. Cabinets (small outpatient facilities) were randomly selected. The intent of the random selection and smaller sample size for cabinets was to gather some initial data on the prevalence and practices of immunization in smaller outpatient services and to guide future research and development of public/private collaborations.

A smaller number of laboratories and pharmacies were selected based on researchers’ assessments of the high likelihood of storage of vaccines at these sites. In terms of clinics, 28 (15.8%) of the 177 licensed and unlicensed clinics in Cambodia were selected. In terms of cabinets, 73 (2.9%) of the 2,499 licensed and unlicensed clinics in Cambodia were selected (8). Table 1 provides an overall picture of sample size and distribution according to location and type of facility.

**Table 1:** Survey sample: private-sector immunization study, 2005*

Facility	Not licensed		Licensed		Applied for license		Total	
	No.	%	No.	%	No.	%	No.	%
Clinic	1	3.6	17	60.7	10	35.7	28	100
Cabinet	7	9.6	51	69.9	15	20.5	73	100
Pharmacy	0	0	15	88.2	2	11.8	17	100
Laboratory	3	33.3	4	44.4	2	22.2	9	100
Location								
Phnom Penh	0	0	45	88.2	6	11.8	51	100
Battambang	3	12	20	80	2	8	25	100
Kampong Cham	3	11.5	13	50	10	38.5	26	100
Sihanoukville	5	20	9	36	11	44	25	100
Total	11	8.7	87	68.5	29	22.8	127	100

*Vaccines provided in the public sector in 2005 included DPT, hepatitis B vaccine, tetanus vaccine, BCG, and measles vaccine

The NIP sent a letter to all selected facilities before researchers arrived. The PATH staff members conducted survey interviews in the presence of government staff. The involvement of the MoH in the study, the NIP letter, and the presence of government staff were necessary to maximize response rate and develop a collaborative research approach. It is possible that the presence of government staff might have biased some survey responses, notably those concerning quality and vaccine leakage (an unauthorized practice in which vaccine earmarked for the public sector is made available at private facilities). Researchers attempted to control for this bias using direct observation methods and by cross-referencing research data with qualitative discussions with private providers on site and in the policy forum. Follow-up site-visits after the policy forum further enabled researchers to confirm key findings, particularly in relation to the issue of vaccine leakage, and to further explore willingness of the private facilities to participate in public-private sector collaborations.

## RESULTS

Results are divided into two sections: the first section presents results from research findings (scope and quality), and the second section describes the development of a proposed model for collaboration between the public health sector and the private-health sector.

### Private-sector research

#### Scope of practice

*Prevalence of private-sector immunization service provision:* A high prevalence of immunization service provision was identified in the private sector. In total, 83 (65%) of the 127 facilities surveyed provided some form of immunization services. Sixty-three (76%) of these 83 facilities stored vaccines. As expected, the proportion of facilities that vaccinated was the highest among 26 (93%) of the 28 clinics surveyed. Of the 73 cabinets, 46 (63%) provided vaccinations (Table 2).

**Table 2:** Prevalence of immunization service provision in the private sector by type of facility (n=127): private-sector immunization study, 2005

Type of facility	Total sample	No. providing immunization services	% providing immunization services
Clinic (inpatient services)	28	26	93
Cabinet (outpatient services)	73	46	63
Pharmacy	17	4	24
Laboratory	9	7	78
Total	127	83	65

Various types of vaccines were available throughout the survey sites, but tetanus and hepatitis B vaccines were by far the most common. Of particular note was the role some clinics played in providing new or underused vaccines (rabies, typhoid, Japanese encephalitis). Table 3 gives an overview of the types of vaccines provided at the surveyed facilities.

**Table 3:** Proportion of private facilities providing selected vaccines (n=127): private-sector immunization study, 2005

Vaccine	No. of facilities	Proportion (%)
Hepatitis B	71	55.9
Tetanus	45	35.4
Rabies	33	26.0
Typhoid	15	11.8
Japanese encephalitis	13	10.2
BCG	12	9.4
DPT	5	3.9
Measles	5	3.9
MMR	2	1.6
Hepatitis A	1	0.8

MMR=Measles, mumps and rubella

Research confirmed that the private clinics and cabinets played a significant role in providing maternal and neonatal healthcare. The same proportions of clinics that vaccinate provided antenatal care services (93%), and slightly fewer conducted deliveries (79%). Of the 24 cabinets that provided antenatal care, 11 reported deliveries in the last 30 days. Seventeen (61%) clinics and 13 (18%) of the 73 cabinets reported 10 or more antenatal care vi- sits in the 30 days preceding the survey. Four clinics and four cabinets reported 50 or more antenatal care visits. Thirteen clinics and four cabinets reported 10 or more deliveries in the last 30 days. Three clinics and one cabinet reported 50 or more deliveries in the last 30 days, highlighting the opportunity that early maternal and child healthcare contacts provided for timely immunization services. These early maternal and child health contacts, in particular, provide an early opportunity for administration of hepatitis birth-dose vaccine, which is most effective if administered within 24 hours of birth. Antenatal care contacts also provide opportunities for neonatal tetanus protection through immunization.

*Source of vaccines and finance*: Vaccines administered by private providers were procured both from government channels and through private suppliers and distributors. Of 202 vaccine procurements within the last 12 months at the 83 facilities that vaccinated, 163 (80%) procurements were sourced from the private sector and 38 (20%) from the public sector. The study, therefore, confirms the movement of vaccines from the public sector into the private sector by unofficial channels (so called ‘leakage’ of vaccine).

The majority of the private providers surveyed procured hepatitis B and tetanus vaccines from the private sector; however, most BCG, measles and DPT vaccines were procured through the public sector. Of the 70 private facilities that administered hepatitis B vaccines, four (6%) procured them from the public sector. However, 15 (more than 33%) of the 45 facilities that administered tetanus vaccines procured them from the public sector. When the private providers were asked as to why they accessed government stock for tetanus vaccine and not hepatitis B vaccine, they indicated that the clients preferred a single-dose presentation of hepatitis B vaccine, and the Government currently does not supply it in this presentation.

Researchers observed evidence of the role international pharmaceutical companies played in the supply of vaccines to private markets in the form of company advertising, distribution, storage, and recording. There was also evidence of vaccination schedule cards being supplied. These immunization cards listed vaccines that were outside the Government's schedule. In some instances, researchers observed posters on clinic walls that listed vaccines and a telephone number for collection of vaccines. The cold-chain and transport systems of the private procurement companies were unknown. There was no formal linkage between the supply of these vaccines and the quality-control system of the Government for procurement, transport, and administration of vaccines.

*Cost for procurement of vaccines.* Survey results demonstrated that cost varied according to the type of vaccine. BCG was the cheapest (median price of US 50 cents) and hepatitis B the most expensive (median price of US$ 7). For most vaccines, the reported costs varied significantly. In the case of Japanese encephalitis vaccine, the price varied from US$ 4 to US$ 19, and in the case of typhoid vaccine, the price ranged from US$ 7 to US$ 15.

The majority (60.6%) of vaccines obtained by the private sector from the public sector were collected free of charge. Of the 15 facilities that reported getting their tetanus vaccines from the public sector, six got them free, while seven paid US$ 1 or more per dose (there were 2 ‘no’ responses). All four facilities that procured hepatitis B vaccines from the public sector got them free; six of nine for BCG; two of three for both DPT and measles.

*Private-sector user-fees for immunization*: Most private facilities that got free vaccines from the public sector charged a fee for administering them. The exception to this case was BCG vaccine, for which five of the six facilities that got it free did not charge for its administration. The vaccines not available in the public sector (hepatitis A, Japanese encephalitis, and measles, mumps and rubella) generated greater average unit profit margins, ranging from US$ 2 to US$ 4 (median unit profit margins from US$ 2 to US$ 3).

### Quality of care and private provider knowledge

*Cold-chain management:* The majority of the vaccines were delivered to the facilities either in cold boxes or in vaccine carriers; however, a significant proportion was transported by regular boxes or other means. Hepatitis B and tetanus vaccines were the two most commonly-transported, stored and administered vaccines. Of the 71 facilities that provided hepatitis B vaccine, 49 (69%) had vaccines transported by a cold box or a vaccine carrier, and 22 (31%) were transported by other means. Of the 45 facilities that provided tetanus vaccine, 34 (76%) had the vaccine transported by a cold box or a vaccine carrier, and 11 (24%) were transported by other means.

*Storage of vaccines:* Almost 90% of facilities that stored vaccines did so in electric refrigerators; however, only one-third monitored the temperature. Only 5% of these facilities had problems in storing vaccines, indicating very low awareness of vaccine-storage requirements and policy.

*Injection safety:* Only 15% of the facilities that stored vaccines used safety boxes to dispose of used needles and syringes, while 83% reported that waste disposal was not a problem. Almost half used other methods for disposal. A comprehensive analysis of these ‘other’ disposal methods is needed to measure the full scope of the problem. The researchers observed unsafe disposal of needles and wastes in ordinary waste receptacles, and some private practitioners indicated that sharps waste was discarded in public waste garbage trucks.

*Health worker's knowledge:* With the exception of some single-dose presentations of hepatitis B vaccine, multi-doses were most common. However, lack of knowledge on how long a multi-dose vial could remain open was commonly reported. Only 28% of the respondents (n=20) were aware of how long they could keep a hepatitis B vaccine vial open, and this figure was only 7% for tetanus (n=3).

Thirty-six percent of the respondents from facilities that vaccinated against tetanus and 11% of the respondents from facilities that vaccinated against hepatitis B reported that they knew at which intervals the successive doses of vaccines need to be administered (Table 4).

**Table 4:** Knowledge of numbers of immunizations per vaccine and intervals for administration (tetanus and hepatitis B vaccines): private-sector immunization study, 2005

Type of vaccination	No.	%
Tetanus vaccination (n=45 facilities)		
Knows correct immunization interval	16	36
Knows correct number of vaccinations	27	60
Hepatitis B vaccination (n=71 facilities)		
Knows correct immunization interval	8	11
Knows correct number of vaccinations	16	23

*Recording of health information:* Sixty-six (80%) of the 83 facilities that vaccinated, kept immunization records. Over half of the facilities that recorded vaccinations did so either on the official MoH card or on their facility's own vaccination card. Thirty-three percent of facilities kept records of vaccination in patient files. There was no evidence that the private-sector clinics systematically reported to the public sector either on immunization numbers, reportable diseases, such as tetanus, measles, and acute flaccid paralysis, or adverse events following immunization.

### Development of a collaboration model

Information on collaboration was sourced from the survey and from the subsequent policy forum where results of the survey were presented by the researchers (see below). The survey respondents expressed interest in all fields of collaboration with slight preferences for training and sharing of information. Management of wastes was the least appealing field of collaboration despite the illustrated dire need for appropriate disposal of syringes and needles.

Most discussion at the policy forum centred on technical aspects of immunization and the mutual roles of the two sectors in financing immunization, particularly in relation to user-fees and provision of cold-chain equipment. It was concluded that more detailed guidelines for regulation and quality improvement of immunization in the private sector should be developed and that these should be negotiated with private clinics on a step-by-step basis.

A summary of quality standards for immunization (Fig.), adapted from the national policy and guidelines, were drafted at the conference. Consensus was reached on the following key points:

- The public sector has a legal responsibility to ensure the quality of care in the private sector through monitoring and professional development support.
- There should be consistency in quality standards between the public and the private sector.
- The public and private sectors should establish health-information communication linkage to ensure that immunizations and related information, such as coverage and disease surveillance, are recorded in a single database.
- All vaccinators in the private sector should have a basic training on immunization.

**Fig.**
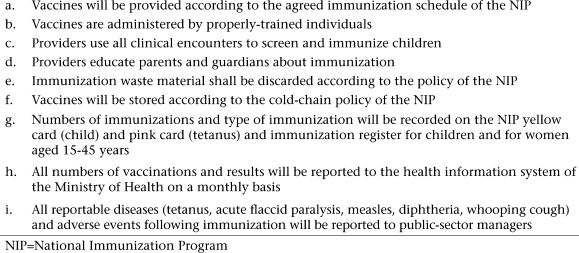
Immunization quality standards: private-sector immunization study, 2005

Guidelines for the implementation of collaboration models were also developed subsequent to policy forum. Key contents included criteria for selecting sites for collaboration (large case load and greater than 30 infant deliveries per month). Implementation steps were defined as the following:

- Conduct a training programme on capacity-building for vaccinators at private facilities.
- Sign an agreement between the MoH and the private-sector manager.
- Provision by the public sector of vaccines to the private sector (tetanus, hepatitis B, and BCG only). This would also involve provision by the public-health sector of auto-disable syringes and waste-management supplies on a monthly basis to the private sector.
- Provision by the public sector of standardized immunization child healthcare cards and selected health-education materials to the private sector.
- Establish a system for reporting the numbers of vaccinations, types of vaccines, adverse events, and reportable diseases to the public-sector manager (to be reported by the private provider).
- Establish waste-management and cold-chain systems that conform to public-sector standards. Refrigerators must be purchased by the private clinics and must conform to the NIP standards. Wastes must be disposed at standard incineration points according to the NIP policy (9).
- Establish an agreement for the monitoring of quality standards through public-sector supervision.

Policy forum consensus was verified at subsequent follow-up meetings with 15 private providers. The private clinic managers reiterated their commitments to establishing a long-term collaboration model based on the above standards and guidelines. At the time of writing, the clinic managers after site consultations with government officials had signed contractual agreements, and activities are now moving from research and development to the implementation phase. On 10 April 2006, the Secretary of State for Health signed into practice guidelines for the implementation of a public-private collaboration for immunization services, based on the quality standards and above implementation steps.

## DISCUSSION

This study resulted in three key findings. First, there was a high prevalence of unregulated immunization service provision in the health clinics in the private sector. Second, important quality issues have been identified in relation to health worker's knowledge, and vaccine and waste management. And finally, both public and private sectors could identify opportunities for strengthening collaboration in the interest of quality improvement.

It is recommended that public-private sector collaborations address the following areas to enhance provision of immunization services: widening access, improving quality, and ensuring affordability and equity.

### Widening access to immunization services through the private sector

One of the opportunities presented by the collaboration is the ability to expand accessibility to immunization services by increasing the number and distribution of service outlets. Public-health advantages can be achieved by expanding collaboration models into rural areas, thus giving rural populations an increased access to preventive health-programme services, such as immunization.

A primary principle of immunization programming is the ‘no missed opportunity’ approach. Opportunistic screening for immunization status during maternal and child health consultations is one example of the implementation of this strategy. The increasing prevalence of births and maternal care in the professional private sector, therefore, provides the added programmatic opportunity to expand the reach of immunization services, particularly in support of elimination of neonatal tetanus and control of hepatitis B (a birth-dose of hepatitis B should be administered within 24 hours of birth). This study has demonstrated that 93% of clinics, which provide maternal and child health services, also provide immunization services. This being the case, there is the potential for the private sector to become a collaborator with the public sector in support of reaching shared public-health goals of elimination of neonatal tetanus and control of hepatitis B.

### Ensuring quality of immunization services in the private sector

One of the important findings of the study was the extent to which the quality of immunization services in the private sector was limited by low levels of health workers’ knowledge in relation to immunization schedules, and vaccine and waste-management practices. This contrasts with the findings of a recent international review of quality of immunization service management and provision in the public sector, which concluded that, although aspects of vaccine management required improvement, it was found that “the overall performance of EPI management at all levels [in the public sector] was relatively good despite financial, human resources and geographical constraints” (7). The same review pinpointed quality problems in the private sector, particularly in relation to management of vaccine, leading to the decision to implement the recommendation to “conduct a review of private sector immunization in Cambodia and develop policy and strategy to improve coverage and quality of immunization services delivered through the private sector by building strategic public and private sector partnerships.” (7)

Clearly, public-private collaborations provide ample opportunities for improving the quality of health services through professional development programmes for private-sector employees and through the establishment of standards for private-sector practice. An important outcome of the policy forum was the agreement between the public and the private sector to establish contractual agreements between facilities and the NIP, specifying the roles and responsibilities of public and private partners in improving immunization service and coverage through the private sector.

### Ensuring equity and affordability of private-sector immunization services

The results of this study indicated that charging a fee for immunization services in the private sector was quite widespread. Consequently, there was a significant discussion at the policy forum in relation to the issue of affordability. It was eventually agreed that the private market should set its own fee for service based on the market value in the area it serves. Additionally, one expectation of the public sector was that the private sector would make its own investments in new cold-chain equipment and waste-management systems.

Through these approaches, the two principles of the free market are met: self-reliance by the market on its own capital investment and freedom to set fees according to the capacity of the market to pay. Affordability is, of course, a relative term, and the capacity of the population to pay within any given catchment area will vary. This highlights the importance of maintaining complementary public-sector services and careful monitoring of both public- and private-sector activities to ensure that high-risk and underserved populations retain access to quality services of preventive health programme, such as immunization.

### Expanding accessibility and quality of immunization services through public-private collaboration

Results of our review of immunization- and the private sector-related literature have indicated low levels of research and evaluation of public and private models of collaboration for delivery of immunization services through the private for-profit medical sector. Most public and private partnerships are conceptualized in terms of government and vaccine manufacturer or government and international agency collaborations. In Cambodia, an evaluation of NGO-contracted health services (as distinct from private for profit medical services) was associated with higher levels of immunization status in children in poorer households than a comparable poor group of children in districts managed by government providers (12). A policy-maker survey of Ministries of Health in the Asian Region has indicated that some viewed a ‘dual channelling’ (i.e. public and private channels) approach to introduction of vaccine as a feasible policy option for introduction of new vaccine (13).

In Cambodia in 2006, private-health facilities (clinics, cabinets [n=2,598]) outnumbered public-health facilities (health centres, hospitals [n=1,041]) by a factor of 2.5 (8). Given increasing rates of urbanization and economic growth in Cambodia in recent years, the trend towards increasing the use of maternal and child health services through the private sector is likely to continue. This provides a stronger case for public-sector immunization managers to adopt a collaborative role with the private sector in support of provision of wider public-health quality service.

Results of this survey indicate that, at the larger private inpatient clinics in Cambodia where maternal and child health services are provided, immunization services are also commonly provided. However, these research findings indicate that these immunization services are also of dubious quality, particularly in relation to the quality of transport and storage of vaccine, management of wastes, limitations in health worker's knowledge of the appropriate time intervals between administration of vaccines, and absence of information exchange (including disease reporting) between the private and the public sector. Further research is needed to ascertain the scope of immunization practices at cabinet outpatient services.

Although the results of the survey have highlighted these significant quality limitations in the private sector, it is considered that public-private collaborations on standard setting of professional development, information exchange, and monitoring will benefit public health in the longer term by addressing these quality limitations. The research also has confirmed that public and private stakeholders are unanimous in their call for a collaborative as opposed to competitive strategy to enhance the quality and coverage of immunization services in the private sector.

This consensus positions the NIP well with regard to future adaptations that address the rapid changes currently underway in the socioeconomic environment. Developments in civil society, rapid urbanization, and the growth of the private sector are likely to apply an increasing pressure on public-sector immunization managers to adopt increased responsibilities as regulators, and not just providers, of quality immunization services in both public and private sectors.

## ACKNOWLEDGEMENTS

The authors would like to acknowledge the services of the staff of Domrei Co. and to the staff and managers of the National Immunization Program, Provincial Health Departments, and Private Clinician Managers for their collaboration in this project.
